# Impact of Graphene Monolayer on the Performance of Non-Conventional Silicon Heterojunction Solar Cells with MoO_x_ Hole-Selective Contact

**DOI:** 10.3390/ma16031223

**Published:** 2023-01-31

**Authors:** Eloi Ros, Susana Fernández, Pablo Ortega, Elena Taboada, Israel Arnedo, José Javier Gandía, Cristóbal Voz

**Affiliations:** 1 Departamento de Ingeniería Electrónica, Universitat Politècnica de Catalunya (UPC), 08034 Barcelona, Spain; 2 División de Energías Renovables, CIEMAT, Avda. Complutense 40, 28040 Madrid, Spain; 3 das-Nano, Polígono Industrial Talluntxe II, Calle M-10, Tajonar, 31192 Navarra, Spain; 4 Departamento Ingeniería Eléctrica, Electrónica y de Comunicación, Universidad Pública de Navarra, Campus Arrosadía, 31006 Pamplona, Spain

**Keywords:** graphene, hole-transport-layer, transition metal oxides, non-conventional silicon heterojunction solar cells

## Abstract

In this work, a new design of transparent conductive electrode based on a graphene monolayer is evaluated. This hybrid electrode is incorporated into non-standard, high-efficiency crystalline silicon solar cells, where the conventional emitter is replaced by a MoO_x_ selective contact. The device characterization reveals a clear electrical improvement when the graphene monolayer is placed as part of the electrode. The current–voltage characteristic of the solar cell with graphene shows an improved FF and V_oc_ provided by the front electrode modification. Improved conductance values up to 5.5 mS are achieved for the graphene-based electrode, in comparison with 3 mS for bare ITO. In addition, the device efficiency improves by around 1.6% when graphene is incorporated on top. These results so far open the possibility of noticeably improving the contact technology of non-conventional photovoltaic technologies and further enhancing their performance.

## 1. Introduction

Graphene is an attractive candidate for a new generation of transparent conductive electrodes (TCEs) with huge impact in different research field domains, such as displays, touch screens, or solar cells, and it is recently setting foot in the commercial market [1,2,3,4]. Graphene, defined as a single layer (monolayer) of carbon atoms, shows unique characteristics that make it a very versatile material. Among them, its mechanical, electrical, and optical properties are considered very attractive for energy-generating devices, which makes graphene a very promising material for near-future energy technology [5,6]. Since its discovery in 2004 by André Geim and Kostya Novoselov at the University of Manchester, several fabrication techniques have been developed and well-established: mechanical exfoliation of highly organized graphite sheets [7], supersonic spray preparation [8], laser-assisted processes [9], or chemical vapor deposition (CVD) [10]. The main limitations and obstacles to integrating graphene in device technologies remain the following (i) the achievement of cost-effective high-quality crystalline graphene; (ii) compatibility of the parameters used during graphene transference; and (iii) scale-up to mass production for covering large areas. These challenges remain open even at laboratory scale [11,12,13]. In this sense, CVD is considered one of the most promising approaches that allows the synthesis of high-quality graphene material in a controllable and reproducible way [14]. Due to the potential benefit of incorporating graphene into devices for various applications, there have been significant efforts focused on developing efficient and reliable transfer methods. This activity has already enabled the successful incorporation of graphene playing in diverse roles as a transparent electrode [15,16], interfacial layer, or an electron acceptor [17,18] in different photovoltaic technologies (i.e., organic, dye-sensitized, and even silicon). However, research on technologies containing graphene in the structure is still at a laboratory scale, and more efforts are needed to implement it into the chain production.

Nowadays, the PV market continues to be dominated by crystalline-silicon technology, which requires new non-conventional solutions to reduce costs. In this sense, silicon-heterojunction (SHJ) technology is considered a reliable low-temperature and high-efficiency solution. In this scenario, materials used in other emerging technologies (i.e., transition- metal-oxides (TMOs), or alkaline salts) are being intensively studied as alternative charge-carrier collectors to traditional doped amorphous silicon layers. The main advantages of using these materials include the following: (i) simple deposition techniques at low-temperature; (ii) no hazardous gas precursors are employed; and (iii) less parasitic absorption compared with traditional heterojunctions. In this respect, successful high-efficiency solar cells implementing such materials have already been reported [19,20,21]. 

Regarding the use of graphene in SHJ technology, hybrid concepts have already been studied, demonstrating the possibility to enhance the photovoltaic performance [22,23]. Particularly, graphene layers have been introduced onto conventional transparent-conductive-oxide (TCO) electrodes such as indium-tin-oxide (ITO). This combined structure provides a significant reduction in the device series resistance, which results in a higher fill factor. Furthermore, the TCO continues to play its role as an antireflection coating due to the very high optical transmittance of the graphene layer [24]. If technical issues can be addressed, this solution can definitely improve SHJ solar cells.

**Figure 1:**
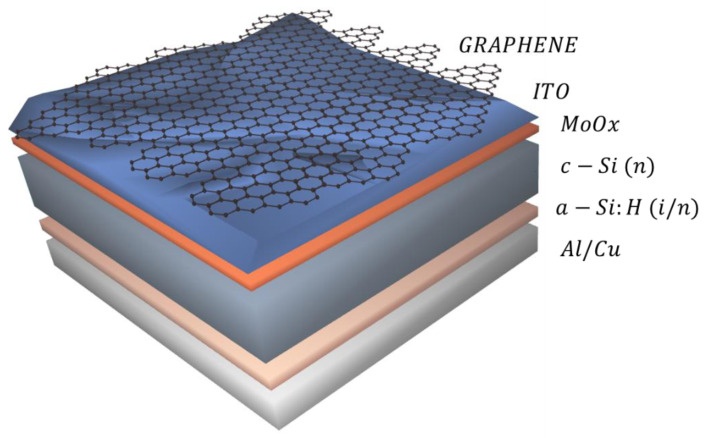
Schematic representation of the fabricated silicon heterojunction solar cell using molybdenum oxide as the hole-selective contact and implementing a graphene monolayer on the front transparent electrode.

In this work, a graphene monolayer is incorporated on the front electrode of a non-conventional crystalline silicon solar cell. Specifically, a 50 nm-thick molybdenum oxide (MoO_x_) hole-selective layer replaced the p-doped amorphous silicon layer of conventional heterojunction solar cells. The graphene monolayers form part of the transparent electrode and are transferred at the end of the fabrication route, just before the last metalization step (Figure 1). The compatibility of the conditions used for graphene transference with the integrity of the solar cell is treated as a key issue. Finally, mechanisms that could explain the improvement in device performance due to graphene incorporation are presented and discussed. 

## 2. Materials and Methods

The graphene monolayers were obtained by chemical-vapor-deposition (CVD) by the Spanish company Graphenea S.L. [25]. The CVD fabrication technique was preferred because it can potentially be scaled up maintaining high-purity and relatively good quality material. The fabrication was carried out on copper foil, using CH_4_ as a precursor, then prepared for transfer with a polymethyl methacrylate (PMMA) coating, and finally transferred to the desired substrate. In the transfer process, environments such as O_2_ plasma, UV-O_3_ activation, and high-temperature annealing processes were avoided. The main reason is that they can negatively affect the device, as non-conventional heterostructures are often less stable [26]. Hence, the temperature used in the transfer process did not exceed 120 °C [27]. Raman spectra were obtained using a Jobin-Yvon LabRam HR 800 system with an Ar excitation laser source emitting at 514 nm. The Raman spectra were used to confirm that graphene monolayers were positively transferred and to validate their quality. 

The solar cells were fabricated on n-type (2 Ω·cm) 280 μm-thick flat c-Si wafers with a (100) orientation. The wafers were first dipped in diluted HF (1%) to etch the native silicon oxide. Then, intrinsic and n-doped amorphous silicon layers (i/n stack) were deposited on the rear side by plasma-enhanced CVD to obtain a good reference electron-selective contact [28]. Following, a 50 nm-thick hole-selective MoO_x_ layer was thermally evaporated on the front side. The MoO_x_ film was coated by an ITO transparent electrode that was much thinner than normal (<20 nm). This stack still worked pretty well as an anti-reflection coating, since the refractive index of MoO_x_ is similar to that of ITO at the wavelength of interest [29]. The ITO coating protects the MoO_x_ layer and also serves as a transparent electrode. Interestingly, it can be kept much thinner because the final sheet resistance of the device will be further reduced by the use of graphene. Consequently, much less indium is needed in this structure compared with standard front transparent electrodes [30]. The rear side was finished by evaporating a 1 μm-thick aluminium contact that was protected by a photoresist before continuing the fabrication route. Furthermore, the next step was already the transference of a graphene monolayer on the front side following a procedure developed in a previous work [27]. Next, solar cells of 1 cm^2^ and 4 cm^2^ were defined by photolithography. Finally, the devices were completed by evaporating a 2 μm-thick Ag grid contacting 4.5% of the device area. A set of solar cells was fabricated, skipping the graphene transfer step to serve as a reference. 

The current density vs. voltage (J–V) electrical characteristics of complete devices were measured in a four-probe configuration using a 2601B Source Meter (Keithley Instruments, Solon, OH, USA). The J-V curves under standard test conditions (100 mW/cm^2^, AM1.5 g solar spectrum, 25 °C) were measured using an ORIEL 94021A (Newport Corporation, Irvine, CA, USA) solar simulator. The external-quantum efficiency curves (EQE) of the solar cells were measured using a commercial instrument, QEX10 (PV Measurements, Boulder, CO, USA). Quasi-steady-state open-circuit (QSSV_oc_) measurements were acquired with a system made in-house [31]. This technique provides pseudo-J-V curves, eliminating the effect of parasitic series resistance. This information will be valuable to assess any effect after graphene transference other than reducing the sheet resistance. Additionally, the conductance of the transparent electrodes was evaluated directly using a contactless, non-destructive Onyx system from the das-Nano Company [32]. This patented measurement is based on reflection-mode terahertz time-domain spectroscopy (THz-TDS) in a frequency range from 0.1 THz to 5 THz [33]. This system provides a full-area map with information about the electrical properties, the homogeneity, and the quality of the 2D materials and thin films [34].

## 3. Results and Discussion

**Figure 2:**
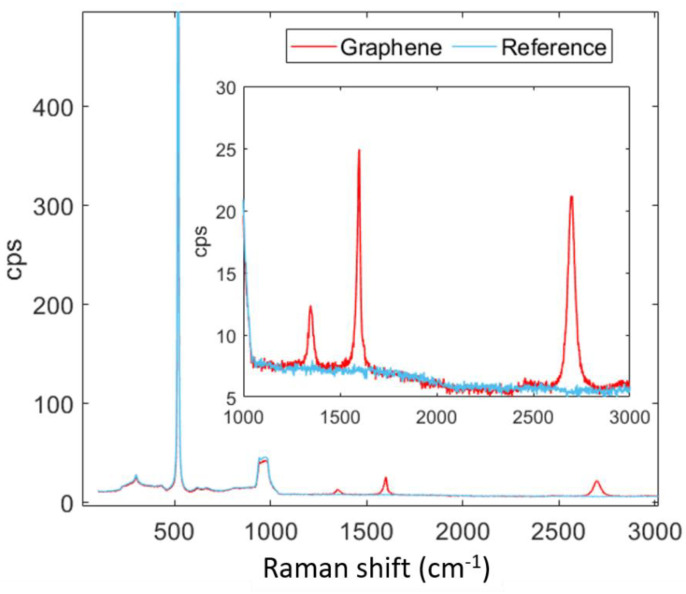
Raman full spectra from 150 cm^−1^ to 3000 cm^−1^ and an inset corresponding to the area of interest with respect to graphene.

After graphene was transferred onto the front electrode, Raman spectroscopy was used as a reliable method to ensure both the presence and quality of the film (Figure 2). The Raman spectrum shows the characteristic peaks expected from high-quality graphene monolayers [35], mainly the G, G * and G’ (also named 2D) bands appearing at ~1590 cm^−1^, ~2450 cm^−1^ and ~2690 cm^−1^. Small signals in the D and D’ bands imply the existence of sp^3^-C defects. Nevertheless, the contribution of both peaks is very small, and defects are possibly localized or due to boundary effects. Furthermore, the presence of sp^3^ hybridized carbon could also be explained by later adsorption due to air exposure or consequence of the wet transfer process. 

**Figure 3:**
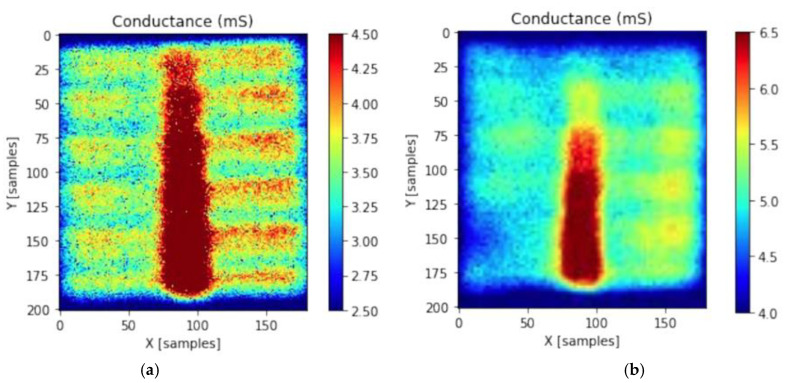
Maps of sheet conductance measured by terahertz reflection spectroscopy for (**a**) reference device (**a**) and with the incorporation of a graphene monolayer (**b**).

Figure 3 shows conductance maps measured by terahertz reflection spectroscopy for a reference solar cell (a) compared with the same device coated with a graphene monolayer (b). The Onyx system from das-Nano is able to resolve the busbar and fingers of the front metallic grid in the refence device [32]. A background sheet conductance of 3 mS is measured on the ITO region, which increases up to 4.50 mS on the busbar (Figure 3a). Adding a graphene monolayer clouded the contrast over the device area, making it harder to distinguish the fingers from the ITO layer underneath (Figure 3b). The sheet conductance of the background is in this case 4.5 mS, which increases to 6.5 mS in the busbar region. The results from this experiment are summarized in Table 1, where normalized values of the series resistance are also shown. Summarizing, the graphene layer positively contributes to reducing the series resistance of the front electrode. The effect is similar to that observed in conventional silicon heterojunction solar cells, where the device performance improved by adding a graphene monolayers [23].

**Figure 4:**
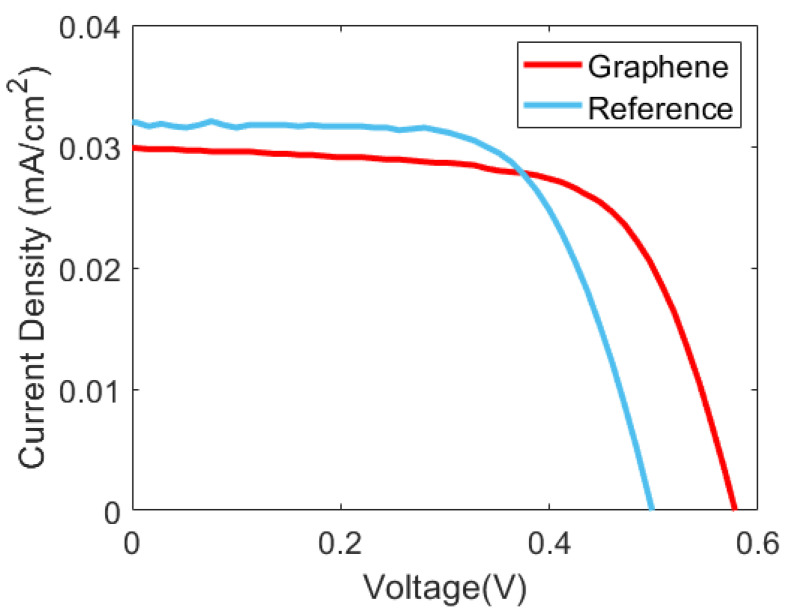
JV curves of the reference and graphene-coated solar cells measured under AM1.5 g illumination (100 mW/cm^2^) at room temperature (25 °C).

**Figure 5:**
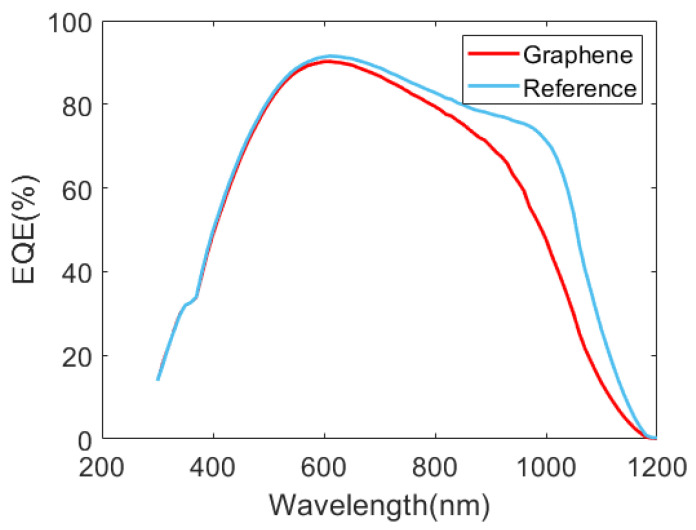
External Quantum Efficiency extracted from the fabricated solar cells.

Further information can be extracted from the current-voltage characteristics (Figure 4) and the External Quantum Efficiency (EQE) curves of the solar cells (Figure 5). Table 2 compares the main photovoltaic parameters of the reference and graphene-coated solar cells, evidencing that this modification clearly improved the final performance. However, there is also a slight reduction in the short-circuit current density (J_sc_) of the graphene-coated cell (31 mA/cm^2^) compared with the reference device (32 mA/cm^2^). This difference could a priori be related to optical absorbance by the graphene layer. However, EQE measurements indicate that the main difference in photocurrent collection is observed at wavelengths between 800 nm and 1100 nm. The behavior in this near-infrared region of the EQE curve is generally associated with rear surface recombination and the quality of the back reflector. A possible explanation is that the rear contact suffered some degradation during the wet transfer of the graphene sheet. The rear side was protected by a thick photoresist to avoid any damage from the reactive material used in this process. Thus, degradation could be related to the thermal step (150 °C) that is also involved in the graphene transference. In order to confirm this, we submitted reference devices to a similar thermal step, and we observed a quite similar degradation (Supplementary Figure S4). This could be understood both as some degradation of the rear surface passivation as well as a decrease in back reflectance. In future work, this degradation can be minimized by intercalating a transparent-conductive-oxide layer between the thin amorphous silicon films on the rear side and the metallic contact.

The slope of the JV curve around short-circuit is also a bit higher for the graphene-coated solar cell, which points to a lower shunt resistance. This effect could be explained by some additional current leakage between the photoactive area of the device and the substrate. Some residues between the devices remained on the graphene-coated substrate after the photolithographic and etching steps were completed to isolate the devices. These can be observed by comparing the device pictures shown in Figures S1 and S2 (Supplementary Information). However, this seems not to be the cause of the lower shunt resistance because current leakage did not reduce after scribing the devices. Some pinholes may be seen on the front electrode of the graphene-coated solar cell (inset of Figure S1). This indicates that we actually have internal connection paths within the active area of the device. Then, this step of the fabrication route would still need some further optimization to minimize this problem. The lower shunt resistance can be limiting the fill factor (FF) of the graphene-coated device, which would partially hide the effect of the reduced front contact resistance. To analyze this further, QSSVoc (Suns-Voc) measurements were completed to obtain pseudo-JV curves of the reference and graphene-coated solar cells (Supplementary Figure S3). The pseudo fill factor (pFF) of the reference solar cell reached a rather good value of 84%, while the pFF of the graphene device only reached 80%. These values of the pseudo fill factor confirm that the reference solar cell was indeed better isolated. Nevertheless, the real FF finally measured in the solar cells was better for the graphene-coated device (Table 2). Definitely, this can be attributed to a reduction in the resistance of the front contact due to the effect of the graphene layer. The total series resistance of the device can be calculated using this equation [36]:

(1) $$R_{s}=\frac{V_{\mathrm{oc}}}{J_{\mathrm{sc}}}\cdot \left(1-\frac{\mathrm{FF}}{\mathrm{pFF}}\right)$$

According to this, the R_s_ value of the reference solar cell is 3.35 $\Omega \cdot {\mathrm{cm}}^{2}$ and it decreases to 2.99$\;\Omega \cdot {\mathrm{cm}}^{2}$ for the device incorporating graphene. These values, calculated from direct electrical characterization (comparing JV and QSSVoc measurements), are the total series resistance of each device. It is observed a very good coincidence with the values deduced from terahertz measurements, which are 3.35 $\Omega \cdot {\mathrm{cm}}^{2}\;$ for the reference and 2.95 $\Omega \cdot {\mathrm{cm}}^{2}$ for the graphene-coated front contact (Table 1). The contactless terahertz reflection spectroscopy only senses the contribution of the front electrode to the series resistance. Then, this result clearly indicates that the rear electrode is contributing much less to the measured series resistance. In the reference device, within experimental accuracy, the series resistance is determined by the front contact. Interestingly, the contribution of the rear contact could be estimated at around 40 $m\Omega \cdot {\mathrm{cm}}^{2}$ for the sample with graphene. It could be argued that the rear contact resistance increased during the wet-process used for graphene transfer. This effect could also be related to the EQE degradation detected in the near-infrared region of the spectrum. 

Finally, the open-circuit voltage (V_oc_) of the graphene-coated solar cell is significantly higher compared with the reference device. This might not be expected, as the series resistance should have no direct influence on the measured open-circuit voltage. There is no current flowing through the solar cell in open-circuit conditions. On the other hand, the V_oc_ value may indeed be related to the quality of the selective contact. Generally, thicker ITO electrodes coat the MoO_x_ layer to provide high lateral conductance and good contact with the metallic grid. However, in this work, the ITO thickness was reduced below 20 nm to investigate graphene as an alternative indium-free transparent electrode. In silicon heterojunction technology, it is known that the electrode work function can significantly impact the band alignment at the corresponding contact [37,38,39]. Something similar may be expected for the non-conventional solar cells studied here. Namely, band alignment at the MoO_x_/silicon interface could be modified by the addition of a graphene layer, given that the ITO electrode is very thin. Actually, it has been reported that graphene improved the quality of MoO_x_ hole-selective contacts for perovskite solar cells [40]. Nevertheless, there is another effect that could also explain the increase in V_oc_ of the graphene-coated solar cell. Excitation of surface-plasmon-polaritons (SPP) on graphene monolayers has been demonstrated [41], and various applications in optoelectronic devices have been reported [42]. Particularly, graphene surface plasmons can significantly increase absorption in the substrate solar cells [43,44]. This plasmonic effect would contribute to the higher open-circuit voltage and could also assist charge-carrier extraction via optical excitation [45]. This seems to be the case here, with graphene positively increasing the V_oc_ by 80 mV compared with the reference solar cell. This, together with the higher FF value, translates into an overall 1.6% increase in the power conversion efficiency (PCE) due to the graphene incorporation.

## 4. Conclusions

The goal of this research was to evaluate the effect of incorporating a graphene layer on the front transparent electrode of n-type silicon solar cells with non-conventional MoO_x_ hole-selective contact. For that purpose, graphene monolayers fabricated by CVD were transferred under conditions compatible with the integrity of these devices. The Raman characterization showed a high quality for the transferred graphene monolayers. A novel contactless electrical characterization by terahertz reflection spectroscopy evidenced a 50% increase in sheet conductance (from 3.0–3.5 mS to 4.5–5.5 mS) by the incorporation of graphene. Consequently, the JV curve of the graphene-coated solar cell shows better V_oc_ and FF values for a very remarkable absolute increase in PCE of 1.6%. Hence, this investigation has identified possible applications of graphene-based electrodes in non-conventional solar cells. Furthermore, this use could also be interesting for applications demanding flexible or transparent electronics betting, on a reduced environmental impact.

## Figures and Tables

**Table 1:** Summary of the sheet conductance measurements by terahertz reflection spectroscopy.

Sample Name	Sheet Conductance (mS)	Sheet Resistance (kΩ)	Series Resistance (Ω·cm^2^)
reference	3.0–3.5	0.3–0.35	3.35
graphene-coated	4.5–5.5	0.2–0.22	2.95

**Table 2:** Main photovoltaic parameters of the reference and graphene-coated solar cells. An absolute 1.6% increase in efficiency was achieved by this modification of the front electrode.

Device	V_oc_ (mV)	J_sc_ (mA/cm^2^)	FF (%)	PCE (%)
reference	498	32	65.7	10.4
graphene-coated	580	31	67.2	12

## Data Availability

The data presented in this study are available on request from the corresponding authors. The data are not publicly available due to industrial participation in this research.

## Supplementary Materials

The following are available online at https://www.mdpi.com/article/10.3390/ma16031223/s1, Figure S1: Photography of the graphene-based solar devices fabricated in this work; Figure S2: Photography of the reference solar devices fabricated in this work; Figure S3: Pseudo-JV curves calculated from QSSVoc characterization for the reference and graphene-coated solar cells studied in this work; Figure S4: EQE curves of reference solar cells before and after being submitted to a thermal step (150 °C) similar to the one involved in the process for graphene transference. There is a decrease in EQE the infrared region similar to the degradation observed in the graphene-coated solar cell.
