# Supplementary material for: Impact of Graphene Monolayer on the Performance of Non-Conventional Silicon Heterojunction Solar Cells with MoOx Hole-Selective Contact
Source: Materials (Basel). 2023 Jan 31;16(3):1223. doi: 10.3390/ma16031223 (PMC9921961; doi:10.3390/ma16031223)
Supplement: Supplementary file 1 [file materials-16-01223-s001.zip › materials-2094095-supplementary.pdf]

materials

Supplementary Materials

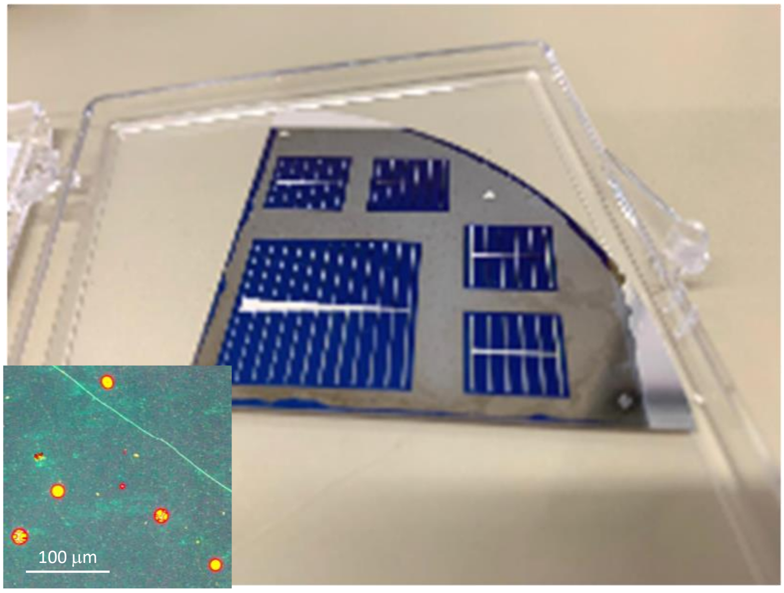

Figure S1. Photography of the graphene-based solar cells studied in this work. The inset shows a detail picture of pinholes observed (optical microscopy) on the front electrode after the graphene transference.

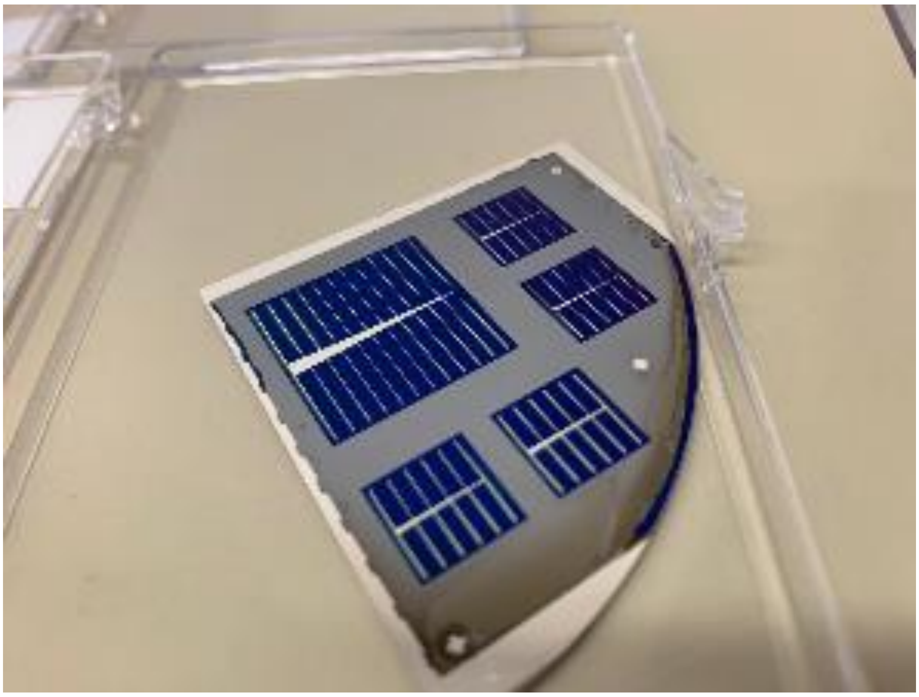

Figure S2. Photography of the reference solar cells studied in this work.

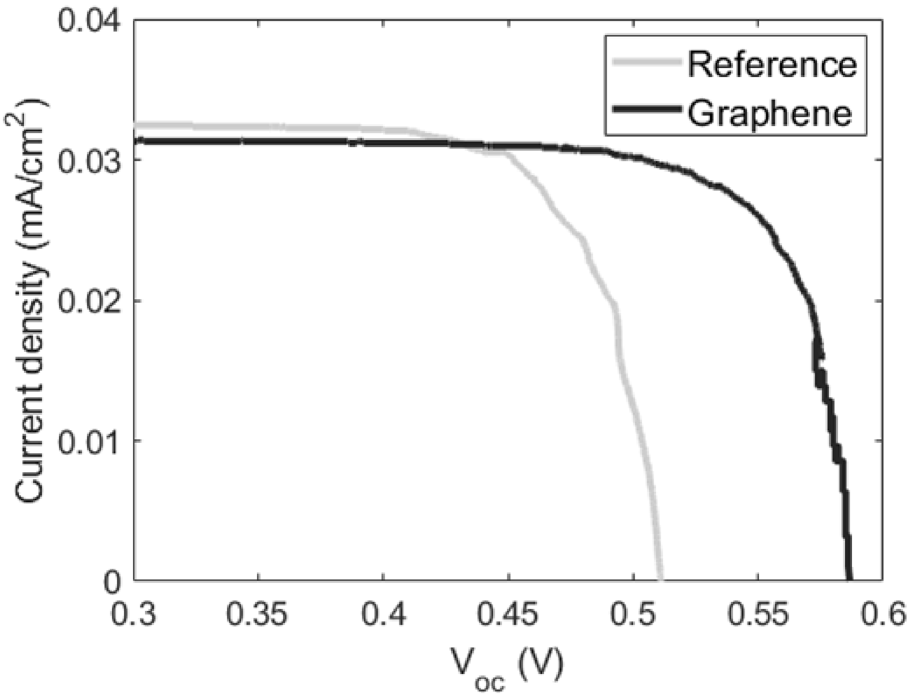

Figure S3. Pseudo-JV curves calculated from QSSVoc characterization for the reference and graphene-coated solar cells studied in this work.

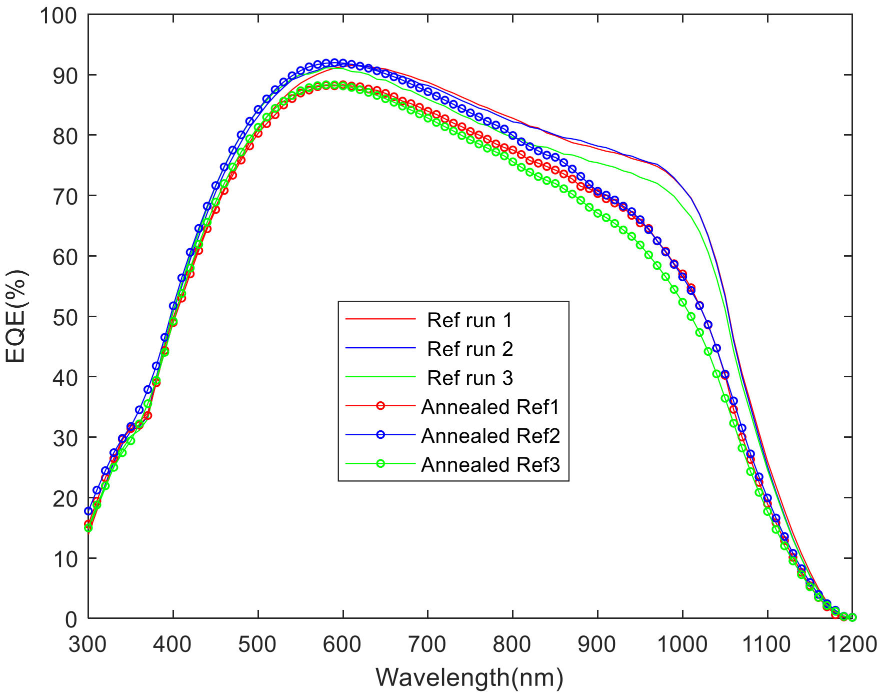

Figure S4. EQE curves of reference solar cells before and after being submitted to a thermal step (150 °C) similar to the one involved in the process for graphene transference. There is a decrease in EQE the infrared region similar to the degradation observed in the graphene-coated solar cell.
